# Prediction model for the compressive strength of green concrete using cement kiln dust and fly ash

**DOI:** 10.1038/s41598-023-28868-7

**Published:** 2023-02-01

**Authors:** Emad S. Bakhoum, Arsani Amir, Fady Osama, Mohamed Adel

**Affiliations:** 1 Civil, Infrastructure Engineering and Management Department, Nile University, Giza, Egypt; 2 Civil Engineering Department, National Research Centre, Giza, Egypt; 3 Contracts Department, ACE Project Management, Cairo, Egypt

**Keywords:** Civil engineering, Mechanical properties

## Abstract

Integrating artificial intelligence and green concrete in the construction industry is a challenge that can help to move towards sustainable construction. Therefore, this research aims to predict the compressive strength of green concrete that includes a ratio of cement kiln dust (CKD) and fly ash (FA), then recommend the optimum sustainable mixture design. The artificial neural network (ANN) and multiple linear regression techniques are used to build prediction models and statistics using MATLAB and IBM SPSS software. The input parameters are based on 156 data points of concrete components and compressive strengths that are collected from the literature. The developed models have been trained, validated, and tested for each technique. TOPSIS method is used to assign the optimum mixture design according to three sustainable criteria: compressive strength, carbon dioxide (CO_2_) emission, and cost. The results of ANN models showed a better prediction of the compressive strength with regression (R) equal to 0.928 and 0.986. The optimum mixture includes CKD 10–20% and FA 0–30%. Predicting the compressive strength of green concrete is a non-destructive approach that has sustainable returns including preservation of natural resources, reduction of greenhouse gas emissions, cost, time, and waste to landfill as well as saving energy.

## Introduction

Construction is one of the main processes of evolution that never stops. Sustainable construction has been a topic discussed in the last century to minimize the negative effect of construction on the environment such as the depletion of natural resources and waste or reuse of any waste material that comes from different industries^1^. Green building is classified under three criteria which are energy, material, and resource conservation. Therefore, a sustainable design is essential and lies under the green building criteria which are related to the material that is used in the design. Eventually, the use of industrial waste material could be the potential solution for energy and material issues. Industrial residues can be utilized as a part of concrete innovations to make eco-friendly concrete.

Concrete has been one of the main materials that are used in almost all structures. The concrete industry causes some environmental problems such as global warming, natural resource depletion, air pollution, and waste. Cement, which is the major component of concrete, is a main producer of CO_2_ and greenhouse gas due to the combustion of fossil fuels for heating the cement-making process, and the thermal decomposition of calcium carbonate for producing cement clinker process. About 88% of the concrete mix emissions are related to cement. Therefore, there is a direct relationship between the CO_2_ emission from concrete production and the cement content used in it. Moreover, cement industry emits a considerable amount of air-pollutant such as Dust, sulfur dioxide (SO_2_), and nitrous oxides (NO_x_). On the other hand, cement production is considered as one of the most energy intensive manufacturing process of all industries^2^.

Consequently, green concrete was used as an eco-friendly concrete. Green concrete is made from waste materials that are produced from construction waste or other industrial wastes. Supplementary cementitious materials (SCM) are those wastes which replacing a portion of cement in the concrete mixture. Hence, reducing the quantities of cement can reduce CO_2_ emissions and air pollution from cement manufacturing. In addition, wastes can be replaced by aggregate, called alternative aggregates, that leads to conserving more natural resources^3^. However, estimating the properties of the produced green concrete is a big challenge because the mechanical properties and durability of concrete is very important. Determining these properties only through laboratory testing consumes a lot of energy and materials besides those tests are destructive approach. Therefore, artificial neural network (ANN) can help to predict the concrete properties in nondestructive approach with saving in energy consumption, materials, and time^4^.

Solid waste management has a significant importance because there is an increase of the generated quantities of by-products. The disposal of industrial by-products is a big concern for industries due to the increasing amount of by-product waste generated and increasing costs of landfills. There are various waste materials and by-products that can be used as replacement of cement in the concrete mixture, such as cement kiln dust, blast furnace slag, fly ash, silica fume, recycled glass, date palm ash, granite waste, and others^5^. Cement kiln dust (CKD) is a by-product waste material resulting from cement manufacturing. Fly ash (FA) is a fine powder that is generated from gases produced by coal-fired electric power generation, and usually discarded in landfills.

This research aims to predict the compressive strength of green concrete that includes a ratio of cement kiln dust (CKD) and fly ash (FA), as industrial by-products, using two methods: artificial neural network (ANN) and multiple linear regression (MLR). In addition, assign the recommended optimum green concrete mixture design based on three sustainable criteria: compressive strength (CS), carbon dioxide (CO_2_) emission, and cost. The methodology of this study can be summarized in the following steps:

- Reviewing of the different research that used CKD and FA as waste materials in green concrete mixtures as well as the different artificial intelligent techniques that are used to predict the compressive strength of concrete.
- Establishing the model database by gathering the necessary information about the components and compressive strength of the concrete mixtures that contain CKD and FA from previous research.
- Applying statistical analysis for the collected data using IBM SPSS software.
- Developing the prediction models using two methods: Artificial Neural Networks (ANN) and multiple linear regression (MLR) using MATLAB and IBM SPSS software. The processes include models training, validation, testing, comparing and analysis of results.
- Performing permutation analysis to get the correlations and importance of input variables of the resulting compressive strength.
- Assigning the recommended optimum green concrete mixture design based on the compressive strength (CS), carbon dioxide (CO_2_) emission, and cost criteria using TOPSIS method as a multicriteria decision making (MCDM) technique.

The novelty of this research is the forecasting of the compressive strength of green concrete mixtures that contain CKD and FA against different combinations of input ingredients (cement, water content, fine aggregate, coarse aggregate, and superplasticizers) in terms of weights. Prediction models are created using two techniques: artificial neural networks (ANN) and multiple linear regression (MLR). Permutation and statistical analysis are performed to evaluate the importance of the input variables on the predicted compressive strength as well as the accuracy of the developed models. The recommended mixture design is assigned after optimization based on technical, environmental, and economic criteria. The benefits of this approach may include the preservation of natural resources, reduction of greenhouse gasses emissions, cost, time, and waste to landfill as well as saving energy.

## Literature review

### Green concrete using cement kiln dust (CKD) and fly ash (FA)

The increasing of green concrete research is due to environmental awareness, economic benefits, and laws^6^. Therefore, several research investigated the properties of green concrete that uses industrial wastes and byproducts. For example, cement kiln dust^7–18^, fly ash^19–22^, blast furnace slag^23–26^, silica fume^27–29^, recycled glass^30,31^, granite waste^32,33^, and Date Palm Ash^34–36^.

Kadhim et al.^16^ used the cement kiln dust CKD that helped filling the micro voids in mixture with proportions of 1, 2, 3, 4 and 5% replacement rations of cement by weight. It improved the mechanical properties such as the compressive strength, direct tensile strength and flexural strength^16^. Omrani and Modarres^17^ studied the effect of CKD and coal waste ash in the emulsified cold recycled mixture. It is concluded that there were high economic and environmental benefit by substituting 1 and 2% of cement content^17^. Yoobanpot et al.^18^ used CKD and the fly ash FA that helped changing the structure of the mortar by adding 10% of CKD & FA as replacement to the same portion of the ordinary portland cement content. The structure was enhanced and densified as the compressive strength developed with time and the voids in the mortar structure were reduced which made it stronger^18^. As mentioned by Abdel-Gawwad et al.^7^ used of CKD and FA helped improving the production of the pozzolan materials as the different reactivity and whiteness were investigated. First materials were treated at 1000 degree then they were used in ratios which affect the strength directly as one of the samples which had fly ash cement kiln dust 80/20 (FA/CKD) produced about 89% whiteness and 100% amorphous content. They replaced 30% by weight of white Portland cement. It resulted in improving the compressive strength after 28 days and enhanced the whiteness^7^.

According to Abdulabbas^8^, the effect of replacing portions of ordinary Portland cement and sulphate resisting Portland cement in concrete mix using certain percentage of cement kiln dust was similar, the substitution of 10% and 20% by weight cement kiln dust as replacement of cement. Another experiment added 10% kiln dust to cement. And finally, another experiment added 20% CKD along with superplasticizer additives. The results showed that the increase of cement kiln dust in the mix and accordingly the decrease in water/cement ratio, the two samples with 10% and 20% additional CKD had increased the compressive strength unlike the replacement samples which showed a decrease in the compressive strength as CKD increased^8^. Al-Rezaiqi et al.^10^ studied the effect of replacing cement by cement kiln dust (CKD) and burnt clay on the concrete properties. It was concluded that replacing cement up to 20% CKD had a non-significant effect on concrete strength and durability^10^.

Strength, segregation, and ease of pumping of concrete can be improved using FA. Using fly ash (FA) from coal-fired power plants has two major benefits. The first is solving the waste problems; the second is reducing overall energy use. The use of fly ash as SCM in various concrete applications has been investigated by many researchers. It can be used as a cement replacement in concrete mix because it contains silica. Fly ash increases concrete strength, improves both sulfate resistance and workability, and decreases both permeability and water ratio required. The utilization of different types of fly ash for artificial sintered aggregate in order to produce lightweight high strength concrete is studied^2,37,38^. Teixeira et al. found that incorporating by-product waste of power/heat production sectors as fly ashes in concrete as cement replacement can improve the environmental performance of the concrete^39^. Bagheri et al.^40^ used different amounts of CKD and FA as a replacement of cement and used Taguchi method to determine the optimal mixing design considering the environmental and economic parameters.

### Prediction modelling for compressive strength of concrete

Recently, many artificial intelligence systems have been widely applied to predict the concrete strength such as neural networks^4,41–44^ and regression analysis^45,46^. Khademi et al.^47^ predicted compressive strength of recycled aggregate concrete at 28 days using three different models which are the (ANFIS) adaptive neuro-fuzzy inference system, (ANN) Artificial neural network, (MLR) Multiple linear regression. It was found that ANN and ANFIS models were better and more accurate than the MLR model and proved that the effect of using the non-dimensional parameters increased the performance of the models^47^. Verma and Kumar^4^, used ANN to forecast the compressive strength of concrete mix including two different cement types: Sulphate resisting Portland cement and Portland cement, and two different types of aggregates: manufactured sand and natural sand, for four different water-cement (w/c) ratios. The predicted strength results were compared to the laboratory results. It was found that the optimum results were obtained for Sulphate resisting Portland cement with manufactured sand^4^.

Bang Ly et al.^44^, proposed a neural network model that to forecast the compressive strength of rubber concrete. Duan et al.^48^ developed an ANN model that can forecast the compressive strength of concrete that includes recycled aggregate instead of natural aggregate. Shahmansouri et al.^49^ constructed an ANN model to predict the compressive strength for geopolymer concrete containing ground granulated blast-furnace slag, silica fume and natural zeolite. The model is verified using different multiple accuracy metrics such as including the mean squared error and correlation coefficient^49^. Chou et al.^50^ proposed an ANN model that can predict the compressive strength of high-performance concrete.

On the other hand, several researchers have worked on developing prediction models using several artificial intelligence techniques to predict the compressive strength of concrete and showed high accuracy towards the prediction of outcomes. For example, using machine learning algorithms of gene expression programming, artificial neural network, ensemble random, and support vector machine to develop predictive models for compressive strength of concrete containing fly ash, lightweight foamed concrete, high strength concrete, self-compacting concrete, and density^51–54^. Moreover, multi expression programming, firefly algorithm, multilayer perceptron neural networks, adaptive neural fuzzy detection systems, genetic programming, random forest regression, individual and ensemble machine learning approaches are utilized to forecast the compressive strength of concrete, carbon fiber-reinforced polymer confined concrete, lightweight foamed concrete, silica fume concrete, and high-performance concrete including waste materials^55–59^.

All prediction models revealed high-accuracy predictions and consistent behavior. It was also concluded that predicting concrete strength shall be conducted using ANN or other machine learning techniques. Finally, an artificial neural network (ANN) is one of the capable techniques that can predict concrete compressive strength, therefore, it is used by several researchers for that objective. Based on the reviewed literature, most studies developed their predictions to the 28-days compressive strength of concrete. However, the research gap can be defined as there is a lack of studies that predict the strength of green concrete containing CDK or CDK with FA as cement replacement waste materials. Furthermore, most studies used input parameters (W/C ratio or % fine aggregate to coarse aggregate) in their models as ratios. In this study, the input variables are inserted in terms of the weight (kg/m^3^) of concrete to improve the usability of the model.

## Establishment of a database

**Figure 1:**
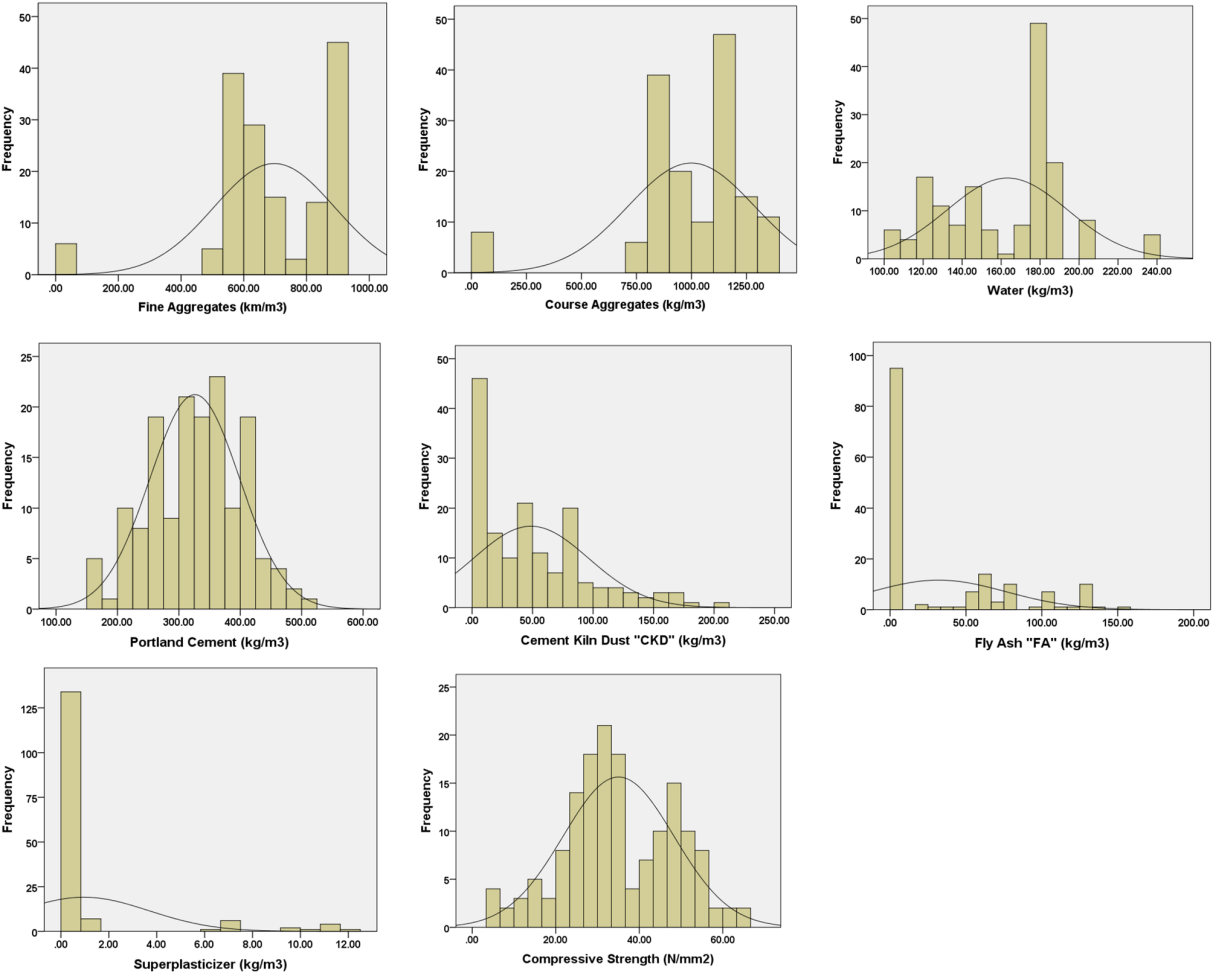
Frequency histogram of variable input parameters and outputs.

This study starts by collecting all experimental data from the literature to establish the database required to develop the prediction model. The database includes 156 data points of green concrete mixtures incorporating CKD and FA, their components, proportions, and resulting compressive strengths. Table 1 lists the range of collected data (fine aggregate, coarse aggregate, water, cement, CKD, FA, superplasticizer, and compressive strength) indicating its sources. The statistical description of the database parameters and the frequency histogram for inputs and outputs are prepared using IBM SPSS software and presented in Table 2 and Fig. 1. The replacement ratios of CKD and FA in all concrete mixtures are 0–50%, and 0–35% respectively.

**Table 1:** Data collection for green concrete incorporating CKD and FA.

Source (Ref.)	Replacement CKD & FA %		Components of the mixes							Compressive strength (N/mm^2^)
			Fine aggregate (kg/m^3^)	Coarse aggregate (kg/m^3^)	Water (kg/m^3^)	Cement (kg/m^3^)	CKD (kg/m^3^)	Fly Ash (kg/m^3^)	Super plasticizers (kg/m^3^)	
^8^	CKD 10, 20%	Min	505	1365	163	324	0.00	0.00	0.00	10.86
		Max	505	1365	190	405	81	0.00	0.01	44.85
^9^	CKD 5, 10, 15%	Min	700	1000	200	327.25	0.00	0.00	0.00	20.40
		Max	700	1000	200	385.00	57.75	0.00	0.00	23.43
^11^	CKD 10, 20, 30, 40, 50%	Min	0.00	770	122.4	170	0.00	0.00	0.00	5.47
		Max	550	1320	122.4	340	170	0.00	1.21	16.22
^13^	CKD 5, 15, 20%	Min	656	1222	182	338.6	0.00	0.00	0.00	33.64
		Max	656	1222	182	423.25	84.65	0.00	0.00	41.47
^14^	CKD 2, 4, 6, 8, 10, 12, 14, 16, 18, 20%. FA 30%	Min	665.78	1152.6	191.5	212.86	0.00	127.7	0.00	25.10
		Max	665.78	1152.6	191.5	425.74	85.14	127.7	0.00	47.80
^15^	CKD 10, 20, 30, 40%	Min	675	950	240	240	0.00	0.00	0.00	12.00
		Max	675	950	240	400	160	0.00	0.00	25.00
^40^	CKD 5,10, 15, 20, 30, 40% FA 0–30%	Min	860	860	111.60	248	0.00	21	0.00	28.09
		Max	915	915	186.11	503	201	151	1.63	55.93
^60^	CKD 10, 20, 30%	Min	893	842	133	315	0.00	0.00	11	46.00
		Max	893	842	133	450	135	0.00	11	65.00
^61^	CKD 10, 20, 30, 40%	Min	841	841	180	240	40	0.00	6	22.00
		Max	867	867	180	360	160	0.00	12	27.50
^62^	CKD 5, 10, 15%	Min	580	1192	200	340	0.00	0.00	0.00	34.53
		Max	580	1192	200	400	60	0.00	0.00	36.29
^63^	CKD 25%	Min	857	853	183	320	80	0.00	9.60	23.78
		Max	857	853	183	320	80	0.00	9.60	23.78
^64^	CKD 5, 10, 15, 20, 30%	Min	733.2	1017.9	105.3	273	0.00	0.00	0.00	38.00
		Max	733.2	1017.9	105.3	390	117	0.00	0.00	56.00
^65^	CKD 5,10, 15, 20, 25, 30%	Min	742.35	0.00	109.87	150.3	0.00	0.00	0.00	23.00
		Max	813.07	0.00	138.27	215.3	38.42	0.00	0.00	36.00
^66^	CKD 10, 20, 30, 40%	Min	665	1232	175	210	0.00	0.00	0.00	15.00
		Max	665	1232	175	350	140	0.00	0.00	26.50
^67^	CKD 5,10, 15, 20, 25% FA 0–35%	Min	580	1192	180	260	0.00	60	0.00	24.40
		Max	580	1192	180	400	99	126	0.00	36.10

**Table 2:** Descriptive analysis of database parameters.

Statistics measures	Input parameters							Output parameters
	Fine aggregate (kg/m^3^)	Coarse aggregate (kg/m^3^)	Water (kg/m^3^)	Cement (kg/m^3^)	CKD (kg/m^3^)	Fly ash (kg/m^3^)	Super plasticizers (kg/m^3^)	Compressive strength (N/mm^2^)
Count	156	156	156	156	156	156	156	156
Mean	697.04	999.35	163.21	325.92	48.72	31.90	0.95	35.03
Std. Error of Mean	15.43	23.02	2.47	5.87	3.81	3.57	0.22	1.06
Median	671.60	1010.74	175.79	331.13	39.75	13.34	0.003	33.22
Mode	580.00	1192.00	180.00	260.00	0.00	0.00	0.00	31.00
Std. deviation	192.68	287.49	30.84	73.32	47.54	44.54	2.72	13.27
Variance	37126.31	82652.92	951.22	5375.51	2259.61	1983.81	7.38	175.99
Skewness	− 1.611	− 1.984	− .032	− .134	.987	1.036	2.961	.008
Std. error of skewness	0.194	0.194	0.194	0.194	0.194	0.194	0.194	0.194
Kurtosis	4.263	5.056	− 0.363	− 0.477	.464	− 0.298	7.458	− 0.457
Std. error of kurtosis	0.386	0.386	0.386	0.386	0.386	0.386	0.386	0.386
Range	915.00	1365.00	134.70	352.70	201.00	151.00	12.00	60.17
Minimum	0.00	0.00	105.30	150.30	0.00	0.00	0.00	5.47
Maximum	915.00	1365.00	240.00	503.00	201.00	151.00	12.00	65.64
Sum	108738.62	155898.80	25461.24	50843.77	7600.71	4976.90	148.55	5465.28

## Prediction Models

In this study, two prediction tools are used separately—to prepare the prediction model of the compressive strength of green concrete to compare their results. Firstly, Artificial neural networks (ANN) technique is selected because it is a computing system that simulates the function of the neurons in the human brain. It works as a self-learning system that helps it to solve complex problems. Just like the human brain, Artificial neural network, contains numerous artificial neurons which are the processing units that is being joined by links, these are connected in the form of a web which process the input and the output information^68^. The general architecture of neural network is made of inputs layer, hidden layers, and output layer^69^. The second tool is the multiple linear regression (MLR) that fits a straight line that minimizes the discrepancies between predicted and actual output values. It can estimate the level of correlation between on dependent variable (compressive strength) from independent variables (input mixture components). It is relatively simple and provides an easy-to-interpret mathematical formula that can generate predictions and compare them to the results of the ANN models. MATLAB and IBM SPSS software have been used to apply ANN and MLR prediction models due to their user friendliness and power.

**Figure 2:**
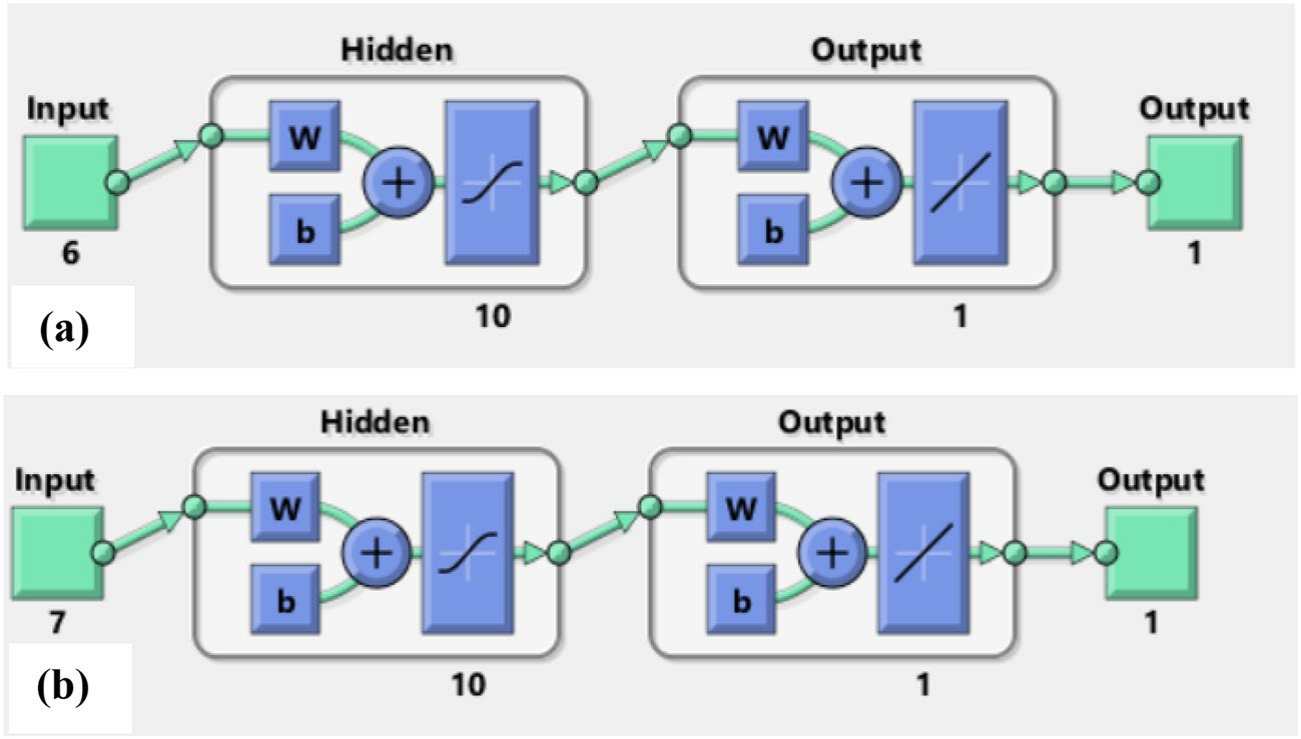
The architecture of ANN models. (**a**) Model 1. (**b**) Model 2.

For each tool (ANN and MLR), two prediction models are developed to predict the compressive strength (CS) of green concrete mixture after 28 days of pouring. The first model is developed for the concrete mixture that includes CKD only as partial replacement. While the second model is developed for the concrete mixture that includes CKD and FA as partial replacement. Both ANN models are trained with different layers to get the optimum number of hidden layers that produce the best regression graphs and prediction results to be used in the final models. The output for each model is similar, which is prediction of the concrete compressive strength. Model (1) includes six inputs representing the components of the green concrete mix including fine aggregate, coarse aggregate, water, cement, CKD, and superplasticizer. While model (2) includes seven inputs representing the components of the green concrete mix by adding FA to the six components of model (1) mixture. Training, testing, and validation samples for each model include 70%, 15%, and 15% of the model data respectively. Figure 2 shows the architecture of ANN for each model.

**Figure 3:**
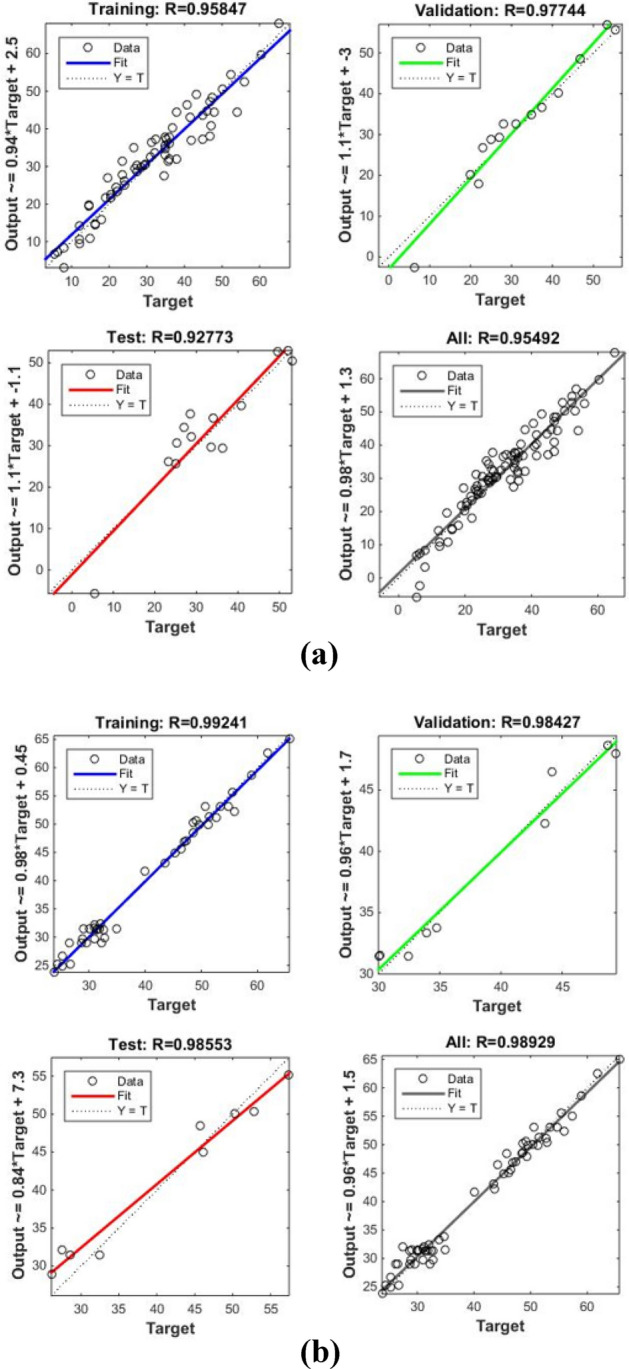
Regression Graphs for ANN models. (**a**) Model 1. (**b**) Model 2.

The ANN is trained to recognize data in patterns. During this process the artificial neural network compares the actual output with the estimated then it adjusts network weights by the difference between both outputs using the backpropagation, after the adjustment, the network produces the lowest error that could occur. Regression graphs for both ANN models are generated and presented in Fig. 3. The graphs show plot between the original output (target) and the estimated output of the training, validation, and training which tends to 1. Values of regression (R) for training, validation, testing, and all are 0.958, 0.977, 0.928, and 0.955 respectively for model (1), and 0.992, 0.984, 0.986, and 0.989 respectively for model (2). The closer the value of R to 1 indicates the better regression.

**Figure 4:**
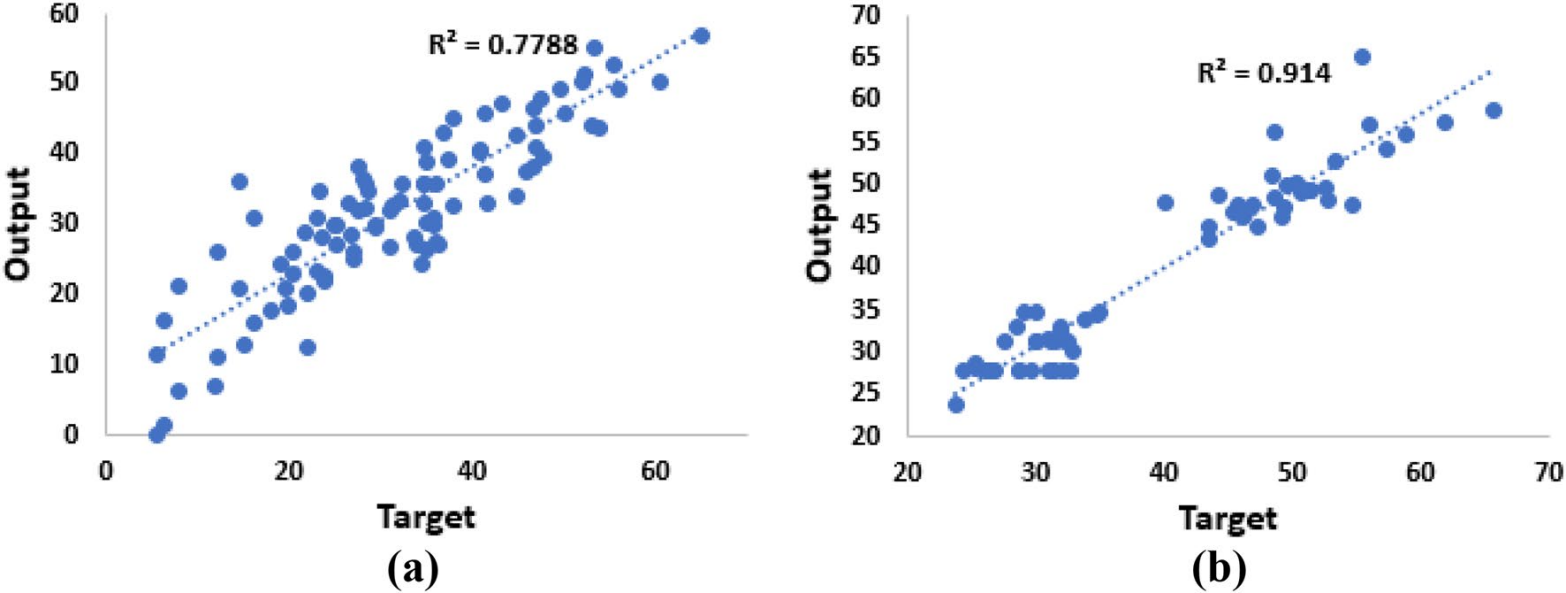
Regression Graphs for MLR models. (**a**) Model 1. (**b**) Model 2.

The models that were built using MLR have similar inputs and outputs to the ANN models. Model (1) has six inputs and one output; Model (2) has seven inputs and one output. The model selection method for MLR is Forward Stepwise. Figure 4 shows that R for models 1 and 2 are 0.78 and 0.91 respectively. In addition, the means for significant effects of input variables on the compressive strength are estimated and found that cement, fine aggregate, and coarse aggregate have positive effects on the compressive strength, while water has negative effect on the compressive strength.

## Results and analysis

**Figure 5:**
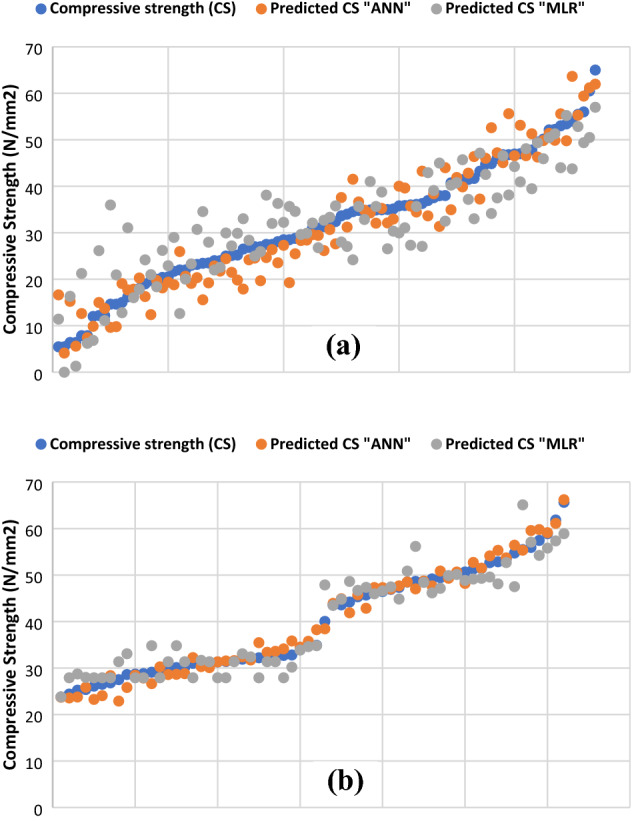
Comparison between predicted and actual compressive strengths. (**a**) Model 1. (**b**) Model 2.

**Figure 6:**
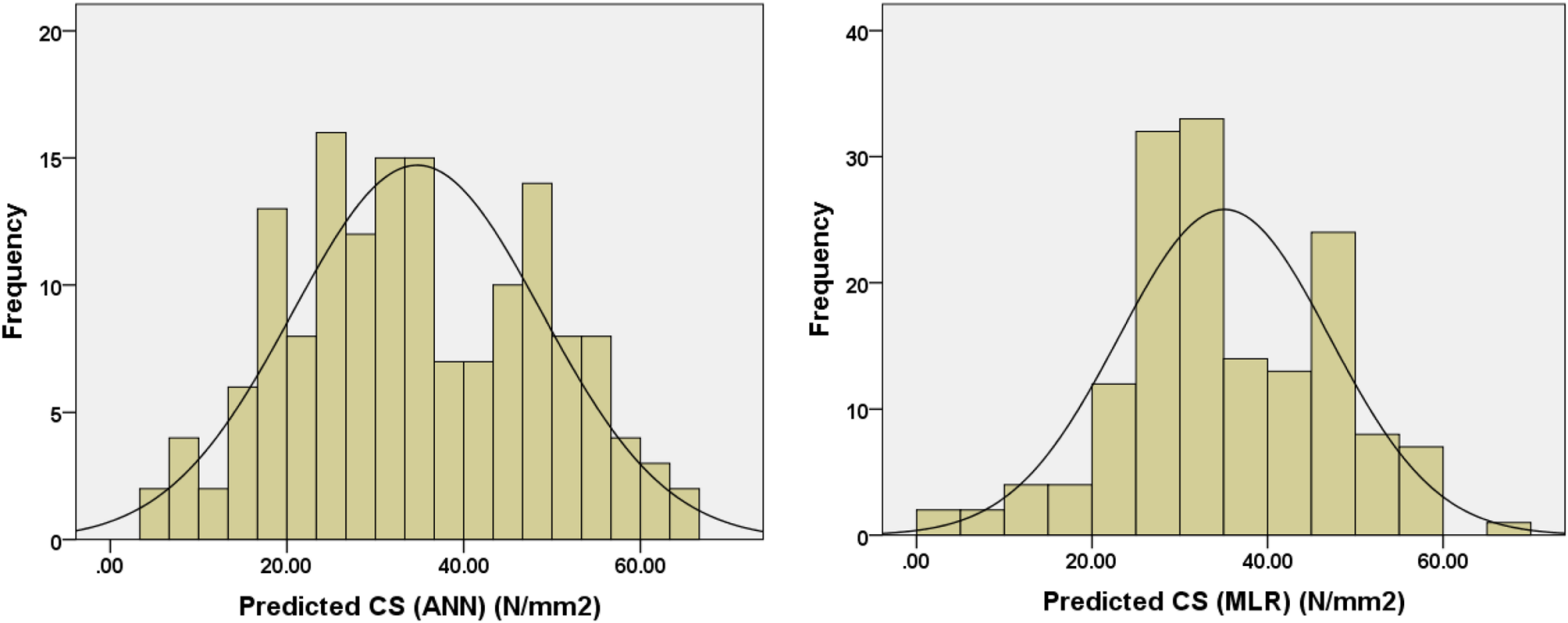
Frequency histograms for the predicted compressive strength (ANN & MLR).

Figure 5 presents a comparison between the predicted compressive strength of ANN models, MLR models, and the actual compressive strength for all the collected data points. The samples are arranged ascending based on its actual compressive strength on the X-axis. The statical description as well as the histograms of the predicted compressive strength using ANN and MLR is presented in Table 3 and Fig. 6. The mean square errors (MSE) for the ANN model 1 are 3.44, 11.20, and 31.30 for training, validation, and testing respectively, and for model 2 are 1.28, 3.80, and 12.40 for training, validation, and testing respectively as presented in Table 4. It is noticed that model 1 has accuracy less than model 2. On the other hand, the ANN models have more accuracy than MLR models.

**Table 3:** Descriptive analysis of predicted compressive strength.

Statistics measures	Predicted compressive strength (N/mm^2^) using ANN	Predicted compressive strength (N/mm^2^) using MLR
Count	156	156
Mean	34.7385	35.0570
Std. error of mean	1.12873	0.96493
Median	33.1652	33.0461
Std. deviation	14.09779	12.05194
Variance	198.748	145.249
Skewness	0.133	− 0.160
Std. error of skewness	0.194	0.194
Kurtosis	− 0.793	0.076
Std. error of kurtosis	0.386	0.386
Range	62.08	65.10
Minimum	4.12	0.00
Maximum	66.20	65.10
Sum	5419.21	5468.89

**Table 4:** Percentage, MSE, and R for ANN processes.

Process	%	Model 1		Model 2	
		MSE	R	MSE	R
Training	70	3.44	0.958	1.28	0.992
Validation	15	11.2	0.977	3.8	0.984
Testing	15	31.3	0.928	12.4	0.986

## Permutation analysis

Seven parameters are employed as inputs: fine aggregate, coarse aggregate, water, cement, cement kiln dust (CKD), fly ash (FA), and superplasticizers (SP). Permutation analysis is performed to determine the most influential parameters affecting the compressive strength of concrete. Table 5 lists the correlations between the input parameters and the predicted compressive strength (ANN models) using Pearson Correlation. It can be noticed that in model 1, there are high positive correlations for cement and fine aggregate. In model 2, there are high adverse correlations for water and high positive correlations for fine aggregate and cement.

**Table 5:** Correlations between the input variables and the predicted compressive strength.

Input parameters	Pearson correlation (compressive strength)	
	Model (1)	Model (2)
Fine aggregate	0.525**	0.583**
Coarse aggregate	− 0.024	− 0.432**
Water	− 0.095	− 0.727**
Cement	0.578**	0.497**
CKD	− 0.250*	− 0.405**
FA	–	− 0.250*
Superplasticizers	0.169	0.393**

**Correlation is significant at the 0.01 level (2-tailed).

*Correlation is significant at the 0.05 level (2-tailed).

**Figure 7:**
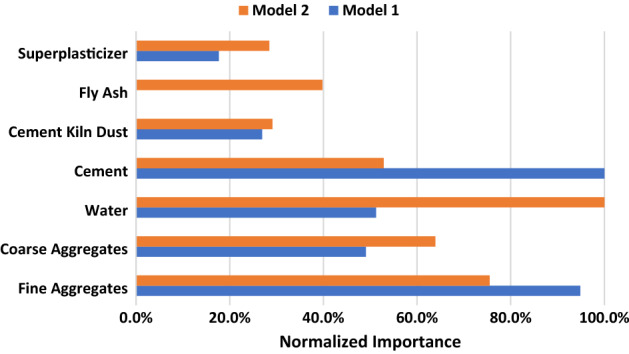
Normalized Importance of variables in the predicted compressive strength.

In addition, ANN models are created using IBM SPSS to validate the results and get the contribution of each variable in the predicted compressive strength. Sum of squares error of are 1.847 and 1.0 (for training), and 3.228 and 0.194 (for testing) for models 1 and 2 respectively. The relative errors are 0.059 and 0.047 (for training), and 0.193 and 0.028 (for testing). The importance of each input parameter to the construction of ANN models is presented in Table 6. Figure 7 presents the normalized importance of inputs showing that that for model 1 the cement has the highest importance followed by the fine aggregate, while for model 2 water has the highest importance followed the fine aggregate. It can be seen in that CKD contributes by 26.9–29.1% in concrete compressive strength prediction.

**Table 6:** Variable importance.

Variables	Importance		Normalized importance	
	Model (1)	Model (2)	Model (1) (%)	Model (2) (%)
Fine aggregates	0.279	0.194	94.9	75.5
Coarse aggregates	0.144	0.164	49.1	63.9
Water	0.151	0.257	51.3	100.0
Cement	0.294	0.136	100.0	52.9
Cement kiln dust	0.079	0.075	26.9	29.1
Fly ash	–	0.102	–	39.8
Superplasticizers	0.052	0.073	17.7	28.4

## Optimization

Selecting the optimum percentage of CKD in the mixture and consequently finding the optimum mixture design is a sustainable challenge to produce green concrete. Therefore, the optimization process can be achieved by optimizing technical, environmental, and economic factors to get the recommended sustainable mixture. Maximizing the compressive strength of the mixture reflects the technical factor. Minimizing the cement contents can reflect the environmental factor because cement is the responsible of the most carbon dioxide (CO_2_) emissions. The quantity of CO_2_ emissions from cement manufacturing is 852 kg/ton^70^. Finally, the cost of cement, fine aggregate, and coarse aggregate can reflect the economic factor. The cost of these components is collected from the local market. It is assumed that waste materials have zero cost. Therefore, the optimization of the mixture design is based on three sustainable criteria: compressive strength, CO_2_, and cost. Table 7 lists the descriptive analysis of the considered criteria (compressive strength is excluded as it is stated before).

**Table 7:** Statistics of the CO_2_ and cost data.

Statistics measures	Carbon dioxide CO_2_ (gm/kg)	Cost (LE)
Minimum	144.84	407.54
Maximum	407.26	936.87
Mean	273.62	695.07
Std. error of mean	5.45	10.48
Median	272.64	695.44
Std. deviation	53.09	102.13
Variance	2818.12	10431.05
Skewness	0.073	− 0.077
Std. error of skewness	0.247	0.247
Kurtosis	− 0.51	− 0.25
Std. error of kurtosis	0.49	0.49

Technique for Order Preference by Similarity to the Ideal Solution (TOPSIS) is used as one of the popular MCDM methods. In TOPSIS, the rank of alternative mixtures is calculated according to their separation from an ideal point. The best measure is that has the shortest distance to the positive ideal solution and the longest distance from the negative ideal solution. The distance Ri^+^ (similarity or relative closeness the positive-ideal solution) is assigned to each alternative mixture within a range between 0 and 1. The final order is obtained sorting the set of alternatives decreasingly in terms of R_i_^+^^71^. The following steps of TOPSIS method are followed for an alternative i under evaluation criterion j and its weight w_j_:

1 $$ {1}{\text{.}}\,\,\,{\text{Normalization}}.\,\,\,\,{\text{r}}_{{{\text{ij}}}} = \frac{{{\text{x}}_{{{\text{ij}}}} }}{{\mathop \sum \nolimits_{{{\text{i}} = 1}}^{{\text{m}}} {\text{x}}_{{{\text{ij}}}} }}\quad {\text{i }} = 1, \ldots ,{ }\,\,{\text{m}};\quad {\text{j}} = 1,{ } \ldots ,{\text{n}} $$

where x_ij_ and r_ij_ are evaluation and normalized evaluation matrix R (respectively)

2 $$ {2}{\text{.}}\,\,\,{\text{Weighting}}.\,\,\,\,\,{\text{v}}_{{{\text{ij}}}} = {\text{w}}_{{\text{j}}} .{\text{r}}_{{{\text{ij}}}} $$

3a $$ \begin{aligned} & {3}{\text{.}}\,\,\,\,{\text{Positive}}\,\,{\text{V}}^{ + } \,\,{\text{and}}\,\,{\text{negative}}\,\,{\text{V}}^{ - } \,\,{\text{ideal}}\,\,{\text{solutions}}.\,\,\,\, \\ & {\text{v}}_{{{\text{max}}}}^{ + } = {\text{max}}\,\,{\text{v}}_{{{\text{ij}}}} = \left[ {{\text{v}}_{1}^{ + } ,\,{\text{v}}_{2}^{ + } , \ldots ,{\text{v}}_{{\text{j}}}^{ + } ,\,{\text{v}}_{{\text{n}}}^{ + } } \right] \\ \end{aligned} $$

3b $$ {\text{v}}_{{{\text{max}}}}^{ - } = {\text{max}}\,\,{\text{v}}_{{{\text{ij}}}} = \left[ {{\text{v}}_{1}^{ - } ,\,{\text{v}}_{2}^{ - } , \ldots ,{\text{v}}_{{\text{j}}}^{ - } ,\,{\text{v}}_{{\text{n}}}^{ - } } \right] $$

4a $$ \begin{aligned} & {4}{\text{.}}\,\,\,{\text{Separation}}\,\,{\text{measure}}\,\,{\text{from}}\,\,{\text{the}}\,\,{\text{positive}}\,\,{\text{S}}_{{\text{i}}}^{ + } \,\,{\text{and}}\,\,{\text{negative}}\,\,{\text{S}}_{{\text{i}}}^{ - } \,\,\, \\ & {\text{S}}_{{\text{i}}}^{ + } = \sqrt {\sum\nolimits_{{{\text{j}} = 1}}^{{\text{n}}} {\left( {{\text{v}}_{{{\text{ij}}}} - {\text{v}}_{{\text{j}}}^{ + } } \right)^{2} } } { } \\ \end{aligned} $$

4b $$ {\text{S}}_{{\text{i}}}^{ - } = \sqrt {\mathop \sum \limits_{{{\text{j}} = 1}}^{{\text{n}}} ({\text{v}}_{{{\text{ij}}}} - {\text{v}}_{{\text{j}}}^{ - } )^{2} } { } $$

5 $$ \begin{aligned} & 5.\,\,\,{\text{Calculate}}\,\,{\text{R}}_{{\text{i}}}^{ + } \\ & {\text{R}}_{{\text{i}}}^{ + } = \frac{{{\text{S}}_{{\text{i}}}^{ - } }}{{{\text{S}}_{{\text{i}}}^{ + } + {\text{S}}_{{\text{i}}}^{ - } }} \\ \end{aligned} $$

**Figure 8:**
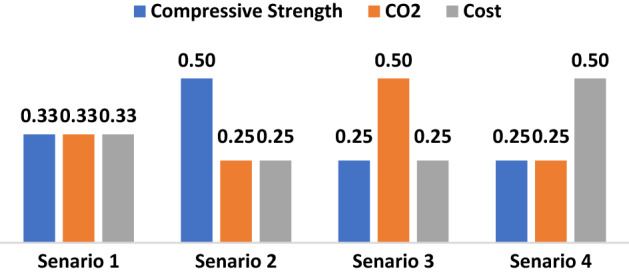
The four scenarios for the relative weights of the optimization criteria.

Bh actual and predicted compressive strengths and considered iividually with CO_2_ and cos to notice and compare results. To assign weights for the three criteria (CS:CO_2_:Cost), four scenarios are assumed as presented in Fig. 8. The first scenario assumes equal weights 0.33:0.33:0.33, the second scenario maximizes the weight of the compressive strength using 50:25:25 ratios respectively, the third scenario maximizes the weight of the CO_2_ using 25:50:25 ratios respectively, and the fourth scenario maximizes the weight of the cost using 25:25:50 ratios respectively.

The optimum mixtures are listed in Table 8 indicating that the optimum mixture includes CKD content from 10 to 20% and FA content from 0 to 30%. It can be seen that for the predicted CS, there is one result for the optimum mixture (20% CKD and 30% FA) for the four scenarios. For the actual CS, there are one different mixture for each scenario with 10–20% CKD ratio and 0–30% FA ratio.

**Table 8:** Optimum Mixtures based on the several scenarios.

	Relative weights (CS:CO_2_:Cost)	fine aggregate (kg/m^3^)	coarse aggregate (kg/m^3^)	water (kg/m^3^)	cement (kg/m^3^)	CKD (kg/m^3^)	fly ash (kg/m^3^)	super Plasticizer (kg/m^3^)	CKD %	FA %
Actual compressive strength										
Scenario 1	0.33:0.33:0.33	733.20	1017.90	105.3	351.0	39.0	0.0	0.0	10	0
Scenario 2	0.50:0.25:0.25	893.00	842.00	133.0	405.0	45.0	0.0	11.0	10	0
Scenario 3	0.25:0.50:0.25	665.78	1152.60	191.5	238.41	59.6	127.7	0.0	14	30
Scenario 4	0.25:0.25:0.50	733.20	1017.90	105.3	312.0	78.0	0.0	0.0	20	0
Predicted compressive strength										
Scenario 1	0.33:0.33:0.33	665.78	1152.60	191.5	212.86	85.14	127.7	0.0	20	30
Scenario 2	0.50:0.25:0.25									
Scenario 3	0.25:0.50:0.25									
Scenario 4	0.25:0.25:0.50									

## Conclusions and recommendations

Green concrete is highly recommended to be used in the construction industry because it has many environmental, technical, and economic advantages. Green concrete can be produced by re-using different wastes in concrete mixtures as cement replacement, resulting in reduction of its negative environmental impacts in addition to increasing its compressive strength. In order to get results of green concrete compressive strength, laboratory works are needed which consumes materials, equipment as well as time and cost. Therefore, the artificial neural networks (ANN) technique and multiple linear regression (MLR) are used to develop models that can predict the compressive strength of concrete including cement kiln dust and fly ash. After that, TOPSIS method is used to get the optimum sustainable mixture design. The developed prediction model is based on the data collected from literature including 156 data points. MATLAB and IBM SPSS software are used to create models and statistical analysis.

Using artificial inelegance applications in construction industry can open the door for development of more innovative approaches that contribute to sustainable construction. Most studies have emphasized increasing the number of samples to enhance ANN training and reduce prediction error. Consequently, samples were developed during training and more than one training model was conducted. Therefore, artificial neural networks can be taught in order to obtain the lowest percentage of error prediction based on trial and error. Training, validation, and testing samples were developed in two models. The first model for green concrete contains CKD. The second model for green concrete contains CKD and FA. It is concluded that ANN model has accuracy better than MLR model. The results of ANN models showed a better prediction of the compressive strength of green concrete with regression (R) equal to 0.928 and 0.986. Finally, TOPSIS method is used to select the optimum mixture design according to three sustainable criteria: compressive strength, carbon dioxide (CO_2_) emission, and cost. It is found that the optimum mixture includes CKD 10–20% and FA 0–30%.

The limitation of the developed model and its accuracy is related to the size and completeness of the collected data from the literature experimental work. This study used data ranges limited to 156 data points. In addition, the study tried to consider similar materials and methods for the collected data, however, the chemical properties of concrete components and the environment of lab experiments for the collected data may be different.

The proposed approach may reflect several advantages including: reduction in CO_2_ and greenhouse gasses emissions by reducing the quantity of used cement; preservation of natural resources by using wase materials; saving in cost and time by predicting the compressive strength without conducting more experimental test on labs, and reduction of waste to landfill by using industrial by-products (CKD and FA) in concrete mix instead of disposal to landfill, and saving energy by replacing cement with waste materials.

The future trends in green concrete applications are various and more research have to encourage its usage. For future research, it is recommended to develop prediction models that focus on other properties of green concrete such as flexural strength, splitting tensile strength, density, workability, resistance to chloride penetration, resistance to sulphate attack penetration, or strength under elevated temperatures. On the other side, prediction models for different concrete mixtures including several wastes such as construction and demolition waste, glass waste, recycled tire rubbers, waste silica, spent foundry sand, steel slag, granulated blast furnace slag, copper slag, marble waste, or quarry dust can be investigated.

## Data Availability

All data generated or analyzed during this study are included in this published article.
